# Quantification of Neuropeptide Y with Picomolar Sensitivity Enabled by Guided-Mode Resonance Biosensors

**DOI:** 10.3390/s20010126

**Published:** 2019-12-24

**Authors:** Mohammad G. Abdallah, Joseph A. Buchanan-Vega, Kyu J. Lee, Brett R. Wenner, Jeffery W. Allen, Monica S. Allen, Susanne Gimlin, Debra Wawro Weidanz, Robert Magnusson

**Affiliations:** 1 Department of Electrical Engineering, University of Texas Arlington, Arlington, TX 76019, USA; mohammad.abdallah@mavs.uta.edu (M.G.A.); joseph.buchananvega@mavs.uta.edu (J.A.B.-V.); kyulee@uta.edu (K.J.L.); 2 Air Force Research Laboratory, Sensors Directorate, Wright-Patterson AFB, OH 45433, USA; brett.wenner@us.af.mil; 3 Air Force Research Laboratory, Munitions Directorate, Eglin AFB, FL 32542, USA; jeffery.allen.12@us.af.mil (J.W.A.); monica.allen.3@us.af.mil (M.S.A.); 4 Resonant Sensors Incorporated, Arlington, TX 76010, USA; gimlin@resonantsensors.com (S.G.); wawro@resonantsensors.com (D.W.W.)

**Keywords:** biomarkers, guided-mode resonance biosensor, neuropeptide Y, optical biosensor, sandwich assay

## Abstract

Assessing levels of neuropeptide Y (NPY) in the human body has many medical uses. Accordingly, we report the quantitative detection of NPY biomarkers applying guided-mode resonance (GMR) biosensor methodology. The label-free sensor operates in the near-infrared spectral region exhibiting distinctive resonance signatures. The interaction of NPY with bioselective molecules on the sensor surface causes spectral shifts that directly identify the binding event without additional processing. In the experiments described here, NPY antibodies are attached to the sensor surface to impart specificity during operation. For the low concentrations of NPY of interest, we apply a sandwich NPY assay in which the sensor-linked anti-NPY molecule binds with NPY that subsequently binds with anti-NPY to close the sandwich. The sandwich assay achieves a detection limit of ~0.1 pM NPY. The photonic sensor methodology applied here enables expeditious high-throughput data acquisition with high sensitivity and specificity. The entire bioreaction is recorded as a function of time, in contrast to label-based methods with single-point detection. The convenient methodology and results reported are significant, as the NPY detection range of 0.1–10 pM demonstrated is useful in important medical circumstances.

## 1. Introduction

Nanopatterned dielectric films provide effective and economic platforms for a host of biological detection applications. The sensor basis is provided by photonic resonance effects originating in lateral leaky Bloch modes propagating along the film. Attendant surface-localized electromagnetic field features enable sensitive biolayer interrogation. Thus, the guided-mode resonance (GMR) sensor operates with quasi-guided waveguide modes induced in the film by incident light [1,2]. The useful resonance signatures are generated in one-dimensional (1D) or two-dimensional (2D) nanopatterns that can be fabricated in large arrays in a reliable, repeatable, and cost-effective manner using nanoimprint methods. Label-free GMR photonic sensors are immune to electromagnetic interference and permit effective light input and output, yielding compact architectures and effective sensing approaches. These sensors can be interrogated with unpolarized white light that excites all allowed resonant modes in the classic orthogonal polarization states where the number of existing modes and associated sensor peaks is controllable by design. GMR sensors exhibit high sensitivity while being arrayable in a compact format and integrated with microwell upper structures in standard formats. Here, we apply this sensor concept and attendant engineered reader system to the detection of neuropeptide Y.

The label-free GMR biosensor was first implemented more than two decades ago. Magnusson and Wang suggested the use of guided-mode resonance for sensor applications and demonstrated optical filters that were tunable by varying the parameters of the resonance structure, including thickness and refractive index [1,2]. Wawro et al. presented new GMR biosensor embodiments, as well as possible applications of these sensors when integrated on optical fiber tips [3]. Refractive index sensing by GMR gratings [4] and use for biochemical assays [5] were subsequently reported. An experiment using a GMR aptasensor showed the capability of real-time, label-free detection of thrombin ranging in concentrations from 0.25–1 μM, with a limit of detection (LOD) of 0.19 μM [6]. GMR biosensors are used for the real-time monitoring of the action of saponin on live cells in the absence and presence of cytoskeleton modulators [7]. In another embodiment, the GMR biosensor consisted of a glass substrate, a waveguide film with an embedded grating structure, and a cell layer used for monitoring ligand-induced dynamic mass redistribution in living cells that were directly cultured on the sensor surface [8]. In general, a major advantage of this sensor methodology is the adaptability to various chemical- and biological-sensing needs via facile modifications of pertinent surface chemistry for any particular application.

Neuropeptide Y (NPY) is the most abundant neuropeptide in the brain; it is a member of a family of proteins that include pancreatic polypeptide, peptide YY, and seminalplasmin [9]. Practical NPY analysis is challenging because NPY exists in the human body at picomolar levels [10,11]; in blood at 0.14–0.6 pM; in urine at 0.1–0.7 pM; and in saliva at 10–12 pM [12,13,14,15]. NPY is associated with stress resilience for the diagnosis of post-traumatic stress disorder, traumatic brain injury, and neurotrauma [16]. Liquid chromatography-mass spectrometry (LCMS) is used to detect NPY in plasma samples, but it exhibits a poor sensitivity, of 5 µM [17]. NPY fluorescence immunoassays demonstrate a sensitivity of 50 pg/mL and a linear range of 0.1–100 ng/mL for NPY; however, this method is time-consuming, requiring a specific clinical environment and instruments to prepare the plate and process the samples [18]. Millipore Human NPY 96-Well Plate Assay (Cat. # EZHNPY-25K) uses fluorescent colorimetry to measure and quantify NPY levels. The standard curve ranges from 5 pg/mL (~2 pM) to 1000 pg/mL (~235 pM).

Recently, an aptamer-functionalized graphene-gold nanocomposite (Gr–AuNs) was used for the label-free detection of dielectrophoretic enriched NPY, where aptamer-NPY binding sufficiently close to the Gr–AuNs surface promotes electron transfer and carries out the electrochemical oxidation of tyrosine. The electrochemical oxidation of tyrosine occurs when NPY molecules bind to the aptamer, while other molecules containing tyrosine do not bind to the aptamer and are incapable of electron transfer. NPY can be detected at 10 pM levels, with linear signal characteristics in the physiologically relevant range of 10–1000 pM NPY [19]. Further, a functionalized graphene field-effect transistor (GFET) device captures NPY directly via bi-domain peptides. A specific biological recognition element P1N3 was used to functionalize GFET devices to produce a biosensor device with picomolar sensitivity to NPY. The GFET shows alteration in the direct voltage for a range of NPY of 1–100 pM [20].

In this research, a guided-mode resonance sensor platform is used to capture NPY using an antibody for rapid and ultrasensitive detection. The platform consists of GMR sensor elements incorporated into the bottom of 96-well microtiter plates, so that a high-throughput assay can be processed instantaneously under the same conditions and with controlled temperature. In concert with the appropriate surface chemistry, NPY is immobilized on the sensor surface. A secondary antibody is added to the well, thus realizing a sandwich assay that enables NPY quantification at sub-picomolar levels. The results presented here show ~x5000 improvement in the LOD over recent preliminary data [21,22].

## 2. Materials and Methods

### 2.1. Materials and Instruments

All analytical grade reagents used in this work were purchased from Sigma-Aldrich (St. Louis, MO, USA), unless noted otherwise. Avidin-D (deglycosylated) was purchased from Santa Cruz Biotechnology (Dallas, TX, USA). The neuropeptide Y (NPY) antibody conjugated with Biotin was purchased from Novus Biotechnology (Centennial, CO, USA). Neuropeptide Y was purchased from Abcam (Cambridge, UK). The sensor plates used were supplied by Resonant Sensors Incorporated (RSI, Arlington, TX, USA). The ResoSens bioassay system, which includes a light source, a temperature control module, and a reader with the Bionetic microarray plates applied here, was manufactured by RSI [23].

### 2.2. Sensor Plate Preparation

**Figure 1:**
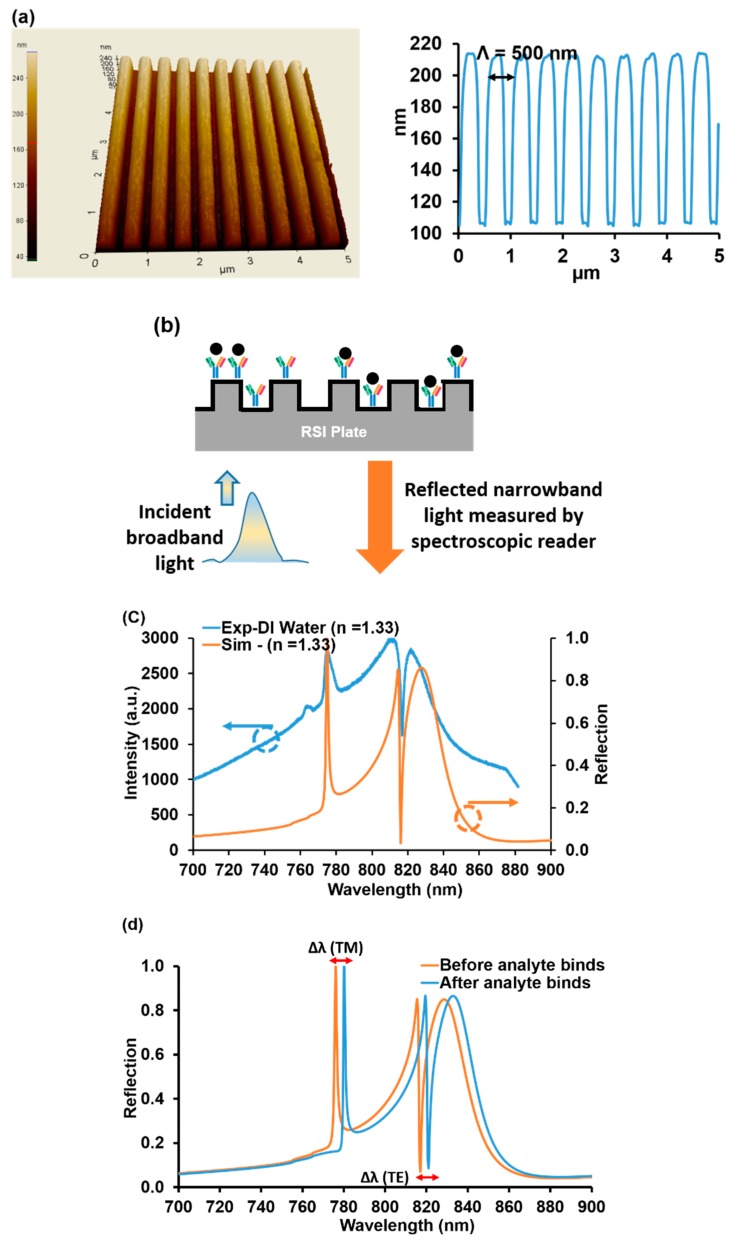
(**a**) 3D atomic force microscope (AFM) image of a guided-mode resonance (GMR) sensor surface with a grating period of ~500 nm and the attendant measured profile. (**b**) Schematic of a GMR sensor operating in reflection mode. Broadband unpolarized light was incident on the sensor in the form of a Gaussian beam. The reflected spectral response was monitored in real-time with an optical spectrum analyzer. (**c**) A measured reflection spectrum of a GMR sensor vs. a rigorous coupled-wave analysis (RCWA) calculated spectrum. (**d**) Calculated reflection spectrum for TM and TE mode resonance by RCWA. Shown is a computed GMR optical biosensor resonance-peak shift due to a change of refractive index from 1.34 to 1.44. In the subsequent sections, these shifts are monitored to quantify the NPY bioreactions.

The biosensors used in this research were fabricated using low-cost submicron molding methods. We employed polymers imprinted with submicron grating patterns (~500 nm grating periods, ~100 nm grating height) coated with a high-index dielectric material (such as TiO_2_) to realize resonant sensors. Figure 1a shows an atomic force microscope (AFM) image of a ~500 nm-period grating and the corresponding measured profile.

Wawro et al. described the sensor measurement methodology, where a broadband light source illuminates the GMR biosensors, and where a specific wavelength of light is reflected (Figure 1b). These sensors are designed to operate in the near-IR wavelength range (700–900 nm), where most biochemical materials have minimal absorption [3]. The reflectance spectral response for TM polarization (magnetic vector normal to the plane of incidence, which is normal to the grooves) and TE polarization (electric vector normal to the plane of incidence) was calculated by a rigorous coupled-wave analysis (RCWA) for the GMR sensor element [24]. Figure 1c shows the reflection spectrum measured for deionized (DI) water and a calculated spectrum using RCWA for a refractive index of 1.33.

The quality (Q) factor can be extracted by calculating the wavelength linewidth ratio (λ/Δλ). The calculated Q-factors for TM and TE peaks were ~130 and ~368, respectively. Notice that the TE “peak” is turned downwards on account of the Gaussian shape of the readout incident light beam. The sensitivity for the TM peak was 107 nm/RIU and for the TE peak 338 nm/RIU. For optical resonance sensors, high sensitivity and Q-factors are desired in order to improve the sensing performance. Circular or tubular-shaped whispering gallery mode optical microcavities are well known to have a narrow linewidth and high Q-factors. Label-free sensing of bovine serum albumin molecules is estimated down to 10 fg/mL [25]. Thus, microring sensor concepts possess high sensitivities but with drawbacks including fabrication and alignment challenges. The GMR sensor technology is attractive based on low cost, easy fabrication, and easy input light coupling, while possessing ample sensitivity and specificity for most applications, as exemplified in this work. The analyte binding changes the local refractive index and therefore the TM and TE resonance peak locations. Moreover, bulk background index variation will affect the resonance positions. Figure 1d illustrates calculated peak location changes for an example bulk refractive-index variation from 1.34 to 1.44. Such GMR wavelength shifts are used to quantify analyte binding that generates local biofilms with finite thickness and density representing a refractive-index increase.

**Figure 2:**
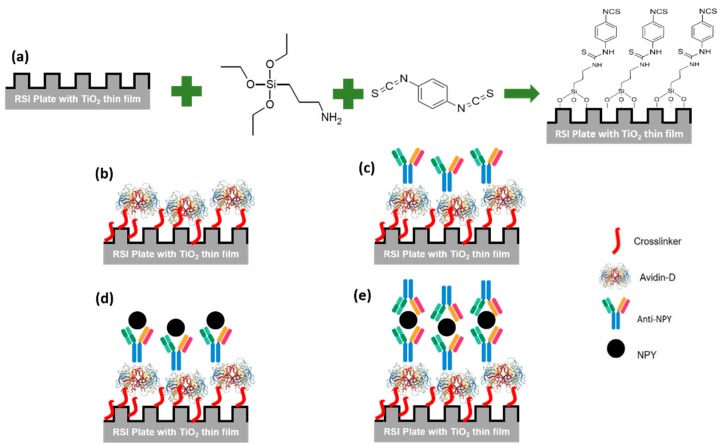
The sandwich neuropeptide Y (NPY) assay principle used in this work. (**a**) Plate preparation for Avidin-D attachment defining cross-linker chemistry. (**b**) Avidin-D immobilization at the sensor surface. (**c**) Covalent coupling of Avidin-D and anti-NPY [Biotin] to form the biorecognition element (BRE). (**d**) NPY attachment to the immobilized BRE. (**e**) NPY sandwich-type detection using secondary anti-NPY molecules.

Proceeding to the experiments, first, the plate was rinsed with ethanol and then washed with deionized (DI) water and dried in N2 flow. Next, the plate was treated with oxygen plasma (PSD Pro-digital UV ozone system) for 10 min to promote the surface hydrophilicity, then it was immersed in 3% (3-Aminopropyl) triethoxysilane (APTES) in a methanol solution for 30 min [26]. The plate was rinsed with methanol and DI water and dried with N2 flow. Subsequently, the plate was baked at 95 °C for 30 min. A dimethylformamide (DMF) solution containing 10% pyridine and 5 mmol/L phenyldiisothiocyanate (PDITC) was prepared, and the plate was immersed in it overnight. The plate was washed with DMF and 1,2-dichloroethane and dried under a stream of nitrogen. Finally, the plate was stored at 4 °C. Figure 2a summarizes this procedure.

### 2.3. Surface Immobilization of Avidin-D

First, we prepared 50, 100, 200, and 250 µg/mL solutions of Avidin-D in phosphate buffer saline (PBS). Next, 50 µL were added to a well and the wavelength shift measured at a controlled temperature of 30 °C to minimize any sensitivity changes due to thermal fluctuations. The wells were washed three times with washing buffer (PBS @ pH 7.4) and measured again to confirm the immobilization of Avidin-D on the surface (Figure 2b). The wavelength shifts of reference wells containing PBS @ pH 7.4 were only measured concurrent with Avidin-D data collection and subtracted from the Avidin-D data to report the net wavelength shift without the background changes (# of wells n = 2). The wavelength shift was plotted vs. time, and the average wavelength shift and standard deviation after the washing step were reported. Then, SuperBlock™ (PBS) blocking buffer purchased from ThermoFisher was added to each well and washed with a washing buffer after 30 min to reduce nonspecific binding on the sensor surface.

### 2.4. Surface Immobilization of Anti-NPY [Biotin]

Solutions of anti-NPY [Biotin] with 20 µg/mL (117.65 nM) and 10 µg/mL (58.82 nM) in PBS were prepared. Next, 50 µL were added in the well, and the wavelength shift was measured at a controlled temperature of 30 °C to minimize any sensitivity changes due to thermal fluctuations. The wells were washed three times with a washing buffer (PBS @ pH 7.4), to remove excess unbound antibodies, and measured again to confirm the coupling of Avidin-D/Anti-NPY [Biotin] to the surface and the formation of the biorecognition element (BRE) (Figure 2c). The wavelength shifts of the reference wells (# of wells n = 2) (immobilized Avidin-D) with PBS @ pH 7.4 were measured concurrent with anti-NPY [Biotin] data collections and subtracted from anti-NPY [Biotin] data to report the net wavelength variation. The wavelength shift was plotted vs. time, and the average shift and standard deviation after the washing step were reported.

### 2.5. Sandwich NPY Assay

The NPY concentration was quantified between two layers of antibodies: the capture and the detection antibody. First, NPY solutions with a final concentration of 0.1 pM (0.4 pg/mL), 0.5 pM (2 pg/mL), 5 pM (21 pg/mL), 62.5 pM (0.3 ng/mL), 125 pM (0.5 ng/mL), 0.5 nM (2 ng/mL), 1.0 nM (4 ng/mL), 5.0 nM (21 ng/mL), and 10.0 nM (43 ng/mL) in PBS @ pH 7.4 were prepared from a 1 µg/mL NPY stock solution. 50 µL were added to the anti-NPY [Biotin] loaded sensor assay wells, and wavelength shifts were measured at a controlled temperature of 30 °C to minimize any sensitivity changes due to thermal fluctuations (Figure 2d). Then, the wells were washed three times with a washing buffer, and 50 µL of the anti-NPY solution (10 µg/mL) were added to the assay wells. Finally, the wavelength shift was measured; then, the wells were washed three times with washing buffer and measured to confirm the capture of NPY (Figure 2e). Again, the wavelength shifts of the reference wells (# of wells = 2) (immobilized anti-NPY [Biotin]) with PBS @ pH 7.4 were measured concurrent with sandwich NPY assay data collection and subtracted from the sandwich NPY assay data. The wavelength shift was plotted vs. time, and the average wavelength shift and standard deviation after the washing step were reported.

## 3. Results and Discussion

### 3.1. Avidin-D Characterization

**Figure 3:**
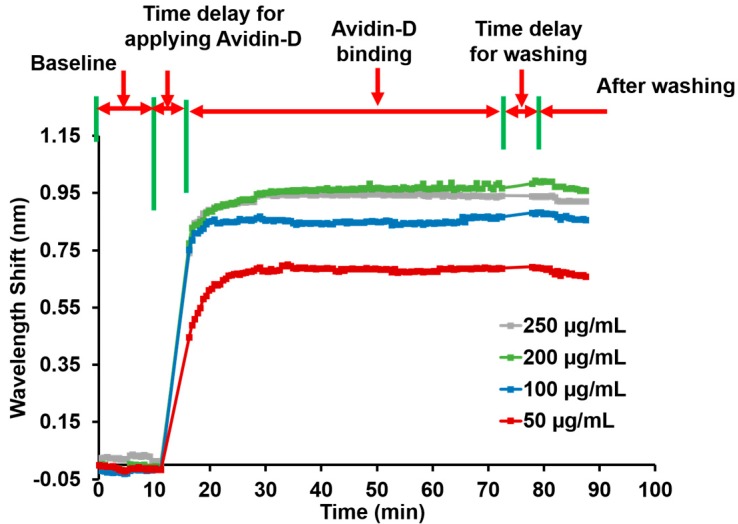
Wavelength shift and process steps as a function of time for 50, 100, 200, and 250 µg/mL Avidin-D concentrations. The final wavelength shifts are approx. 0.67, 0.86, 1.03, and 0.92 nm for 50, 100, 200, and 250 µg/mL Avidin-D, respectively.

The Avidin-D wavelength shift was measured for different concentrations (50, 100, 200, and 250 µg/mL). The 50 µg/mL concentration showed the average wavelength shift after washing ~0.67 ± 0.011 nm. The shifts for 100, 200, and 250 µg/mL of Avidin-D were ~0.86 ± 0.009, ~1.03 ± 0.012, and 0.92 ± 0.008 nm, respectively (Figure 3). The data showed a linear relationship between the Avidin-D concentration and wavelength shift between 50–200 µg/mL. At 250 µg/mL, the wavelength shift declined. Thus, a ~200 µg/mL concentration of Avidin-D suffices to saturate the sensor surface.

Nonspecific binding can be a problem and reduce the sensitivity of the assay. The blocking of remaining active sites on Avidin-D immobilized sensor surfaces with a blocking buffer significantly reduces the nonspecific binding of proteins to the sensor surface, thus yielding lower detection limits.

### 3.2. Anti-NPY Characterization

**Figure 4:**
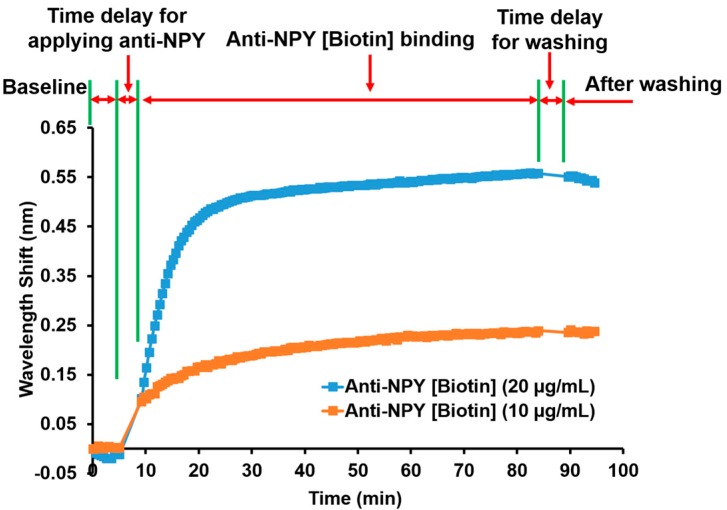
Wavelength shift and process steps as a function of time for anti-NPY [Biotin] binding to Avidin-D. The curves represent triplicate (n = 3) averages.

Avidin-Biotin coupling captures Biotin-tagged anti-NPY on an Avidin-D loaded sensor surface. Figure 4 shows the resonance wavelength shift monitoring for anti-NPY binding to Avidin-D over time. The first step was the baseline measurement to set the reference for all subsequent measurements. Applying anti-NPY produced a resonance wavelength shift that implied a successful binding reaction between the anti-NPY [Biotin] molecules and the Avidin-D layer. The average wavelength shift measured in triplicates was ~0.55 ± 0.005 nm for 20 μg/mL (117.65 nM) of anti-NPY and ~0.24 ± 0.001 nm for 10 μg/mL (58.82 nM) of anti-NPY after the washing step. Avidin-D and Biotin had an equilibrium dissociation constant of ~10–15 M, which produced strong binding affinity, confirming the presence of Biotin-tagged anti-NPY on the sensor surface even with a lower concentration of anti-NPY. The higher wavelength shift for 20 μg/mL anti-NPY showed that more Avidin-D binding sites were available to bind specifically to Biotin-tagged anti-NPY. For the NPY sandwich assay, 10 μg/mL of anti-NPY were used to conduct all the experiments. Further investigation must be completed to find the optimal concentrations and the effect of the BRE concentration on the sensitivity.

### 3.3. Sandwich NPY Assay

**Figure 5:**
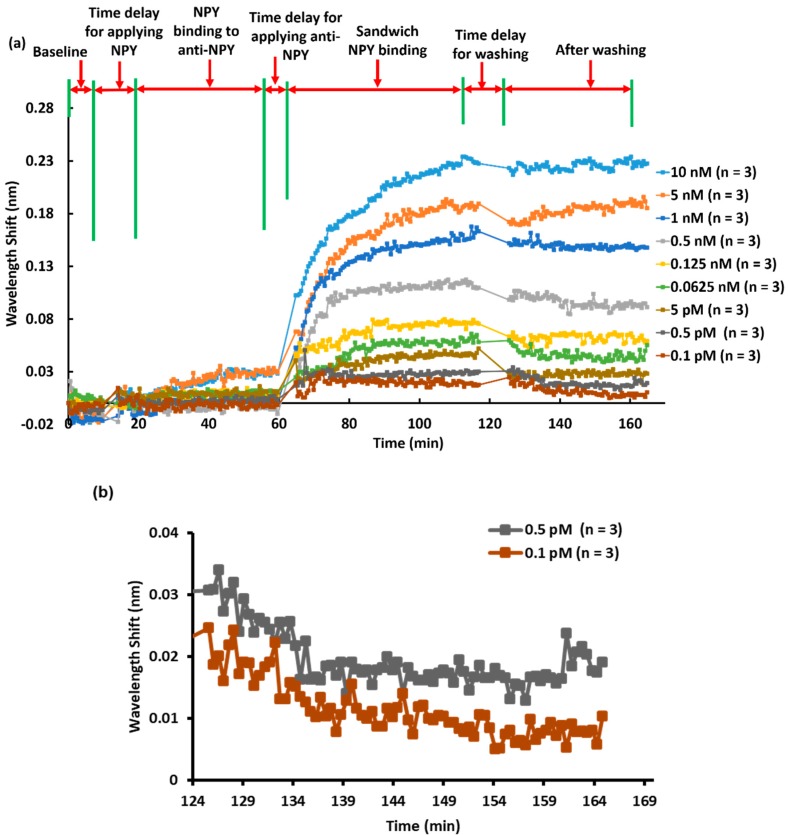
(**a**) Process steps and averaged (in triplicates) wavelength shift response for the NPY sandwich assay as a function of NPY concentration. (**b**) Zoomed-in results for 0.1 and 0.5 pM NPY after the washing step, demonstrating the limit of detection (LOD) pertinent to these experiments.

The anti-NPY/NPY/anti-NPY sandwich assay approach was used to quantify low concentrations of NPY. The process steps are shown in Figure 5a. We applied different concentrations (0.1, 0.5, and 5 pM and 0.0625, 0.125, 0.5, 1.0, 5.0, and 10.0 nM) of the NPY solution to the anti-NPY loaded sensor surface and measured the corresponding resonance wavelength shifts during the ~20–60 min interval; we saw minimal shifts. Next, the second anti-NPY was added to bind to, and detect, NPY over ~60–115 min, as indicated in Figure 5a. We measured averaged wavelength shifts of ~11 ± 5 pm (n = 3), 20 ± 5 pm (n = 3), 27 ± 2 pm (n = 3), 46 ± 3 pm (n = 3), 62 ± 4 pm (n = 3), 96 ± 5 pm (n = 3), 149 ± 3 pm (n = 3), 183 ± 7 pm (n = 3), and 225 ± 4 pm (n = 3) for 0.1, 0.5, 5, 62.5, and 125 pM and 0.5, 1, 5, and 10 nM concentrations of NPY, respectively. Thus, we see that the sandwich assay was extremely successful in detecting miniscule concentrations of NPY. To quantify the limit of detection applicable here in more detail, we focused in on the lowest concentration data. Thus, Figure 5b shows zoomed-in results for 0.1 and 0.5 pM NPY after washing. It is clear that these concentrations provided measurable wavelength shifts and established the final LOD for the class of biomaterials under study.

**Figure 6:**
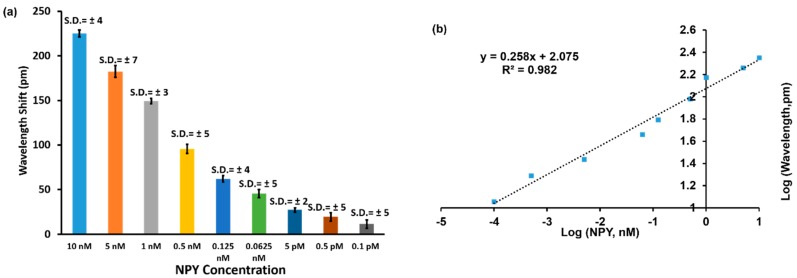
(**a**) Bar chart of wavelength shift vs. NPY concentration. (**b**) Log-log plot of wavelength shift versus concentration. The logarithmic transformation was applied to establish a linear relationship for the concentration range from 0.1 pM to 10 nM of NPY.

Figure 6a shows a bar chart of wavelength shift vs. NPY concentration (n = 3; error bars = ± standard deviation) for the results shown in Figure 5. One-way ANOVA statistical analysis was performed on the wavelength changes for the collected data and showed *p* value < 0.0005, which means the wavelength shifts measured for each NPY concentration were statistically different. The LOD is the lowest amount of NPY concentration that can be detected using the GMR sensor methodology, which was 0.1 pM for NPY in our experiments.

The data were transformed into a logarithmic scale to verify a linear relationship between concentration and resonance wavelength shift. A regression fit was used to estimate the degree of linearity. Figure 6b establishes a linear response of NPY concentrations ranging from 0.1 pM to 10 nM vs. wavelength shift with R^2^ = 0.982. For this data representation, the sensitivity can be expressed as 0.258 Log (Δλ, pm)/Log ([NPY], nM).

## 4. Conclusions

We report the measurement of low concentrations of NPY enabled by the anti-NPY sandwich-type capture of NPY. The initial anti-NPY capture molecules were successfully immobilized on a submicron grating-based sensor surface coated with TiO_2_. We demonstrated a rapid and accurate detection of NPY via the sandwich assay, such that NPY could be detected at levels of 0.1 pM (0.4 pg/mL), which is a ~x20 increase above the Millipore commercialized kit and ~x10 better than the detection limit of functionalized GFETs. The sensor data analysis for NPY indicated a linear response for NPY concentrations in the range of 0.1 pM–10 nM NPY. The optical resonance sensing method, coupled with the rapid assay technique deployed here, with controlled sample temperature and 96-well plates for high throughput, is likely applicable to other technology areas, including enzymes, anti-fouling surfaces, and nanobodies. Further work is necessary to use this approach to detect NPY from human samples and in clinical settings; such experiments are beyond the scope of the current report.
